# Characterization of a transgenic mouse model exhibiting spontaneous lung adenocarcinomas with a metastatic phenotype

**DOI:** 10.1371/journal.pone.0175586

**Published:** 2017-04-18

**Authors:** Hsuen-Wen Chang, Zih-Miao Lin, Min-Ju Wu, Li-Yu Wang, Yen-Hung Chow, Shih Sheng Jiang, Hui-Ju Ch’ang, Vincent HS Chang

**Affiliations:** 1 Laboratory Animal Center, Office of Research and Development, Taipei Medical University, Taipei, Taiwan; 2 The PhD Program for Translational Medicine, College of Medical Science and Technology, Taipei Medical University, Taipei, Taiwan; 3 National Institute of Infectious Diseases and Vaccinology, National Health Research Institutes, Zhunan, Miaoli County, Taiwan; 4 National Institute of Cancer Research, National Health Research Institutes, Zhunan, Miaoli County, Taiwan; University of South Alabama Mitchell Cancer Institute, UNITED STATES

## Abstract

Developing lung cancer in mouse models that display similarities of both phenotype and genotype will undoubtedly provide further and better insights into lung tumor biology. Moreover, a high degree of pathophysiological similarity between lung tumors from mouse models and their human counterparts will make it possible to use these mouse models for preclinical tests. Ovine pulmonary adenocarcinomas (OPAs) present the same symptoms as adenocarcinomas in humans and are caused by a betaretrovirus. OPAs have served as an exquisite model of carcinogenesis for human lung adenocarcinomas. In this study, we characterized the histopathology and transcriptome profiles of a jaagsiekte sheep retrovirus (JSRV)-envelope protein (Env) transgenic mouse model with spontaneous lung tumors, and associations of the transcriptome profiles with tumor invasion/metastasis, especially the phenomenon of the epithelial-mesenchymal transition (EMT). Genetic information obtained from an expression array was analyzed using an ingenuity pathways analysis (IPA) and human disease database (MalaCards). By careful examination, several novel EMT-related genes were identified from tumor cells using RT-qPCR, and these genes also scored high in MalaCards. We concluded that the JSRV-Env mouse model could serve as a spontaneous lung adenocarcinoma model with a metastatic phenotype, which will benefit the study of early-onset and progression of lung adenocarcinoma. In addition, it can also be a valuable tool for biomarkers and drug screening, which will be helpful in developing intervention therapies.

## Introduction

Lung cancer is one of the most common forms of cancer which remains the leading cause of cancer-related deaths worldwide among men and women. Lung cancers are composed of two major histological types: small-cell lung cancer (SCLC) and non-small-cell lung cancer (NSCLC). NSCLCs are further divided into squamous cell carcinomas (SCCs), pulmonary adenocarcinomas (ADCs), and large-cell carcinomas. Among them, lung ADCs are the most prevalent NSCLC. In 2004, the World Health Organization (WHO) classification recognized a particular subtype, bronchioloalveolar carcinoma (BAC), which later was renamed adenocarcinoma *in situ* (AIS), for its non-invasive features and excellent prognosis [1,2]. These non-invasive lung lesions are more commonly found in non-smokers, women, and Asian populations [3,4]. AIS-like areas may also accompany invasive ADCs, referred as mixed-type ADCs with AIS features. AIS is considered the probable cancerous lesion of human lung ADC and develops from its precursor, atypical adenomatous hyperplasia (AAH) [5]. Increasing evidence indicates that AAH is a forerunner of lung ADCs, and AAH and AIS are frequently found adjacent to areas of an invasive ADC, which suggests a multistep process in the development of the invasive phenotype of lung ADC [6]. A recent study showed that genetic alterations involve in the progression of AAH to AIS [7].

The main risk factors for lung cancer include cigarette smoke, asbestos, environmental pollution, and radiation. However, global statistics estimate that 15% of lung cancers in men and 53% in women are not attributable to smoking, overall accounting for 25% of all lung cancer cases worldwide [8]. It was estimated that 15%~25% of human cancers may have a viral etiology, and two viruses, in particular, the human papillomavirus (HPV) and jaagsiekte sheep retrovirus (JSRV), were speculated to play roles in the pathogenesis of human lung cancer [3]. Activation of the PI3K/Akt pathway was reported in various JSRV-transformed cell lines, and it confers a growth advantage [9]. The finding that the JSRV envelope protein (Env) can mediate infection of human cells raised the possibility that JSRV or a related virus might cause cancer in humans [10]. Furthermore, antibodies against JSRV capsid proteins were found to react with about 30% of human BSA and pulmonary ADC samples but not with ADCs from other organs or with normal tissues [11]. In addition, the JSRV is phylogenetically related to human endogenous retroviruses (HERVs), and a structural analysis of the retroviral Env showed that it is highly conserved among most retroviral genera and restricted to the mammalian class [12,13]. However, the recent study [14] revealed that while JSRV can infect human cells, its role in human lung cancer is little.

Wikenheiser *et al* [15] had reported a spontaneous lung tumor mouse model using simian virus 40 large T antigen (SV40) driven by lung-specific promoter alveolar type II surfactant protein C (SPC), which resulted in rapid development of multifocal bronchioalveolar neoplasias. The transgenic mice then died at 4–5 months of age, making the investigation of early events in carcinogenesis difficult. In this study, we used a model utilized a transgene with JSRV-Env in an immunocompetent (FVB/N) background under a lung-specific SPC promoter [16], which developed ADCs in the distal lung epithelium. It was documented to produce spontaneous induction of lung tumors from 1 month of age onwards, with progressive increases in the tumor incidence and tumor size with age. In our mice model, development of lung tumors was slower, with animals living in nearly a year, making it an ideal model for investigating carcinogenesis and cancer prevention.

During tumor progression, the epithelial-mesenchymal transition (EMT) is a process in which epithelial cells gradually acquire a mesenchymal (fibroblast-like) cell phenotype. The EMT is thought to contribute to cancer invasion and metastasis, and treatment resistance [17,18]. NSCLC has a 5-year survival rate of 15%, with metastasis as the primary cause of death, which is much lower than for many other common cancers such as the colon (64.2%), breast (89.2%), and prostate cancers (99.2%) [19]. These features make the EMT a process of high interest as a therapeutic target to improve clinical management of lung cancer. A clear understanding of biological events during the progression of lung ADCs is critical for identifying molecular biomarkers related to carcinogenesis and tumor progression. In this study, we characterized the established JSRV-Env transgenic mouse model with spontaneous lung tumors in aspects of histopathology, transcriptome profiles, and their respective molecular signatures to the progression of human lung ADCs.

## Materials and methods

### Animals and ethics statement

Transgenic FVB mice with the JSRV-Env [16] and spontaneous lung tumors were maintained in independently ventilated cages (IVC) at a constant temperature (20~22°C) and a 12-h light/12-h dark cycle in a specific pathogen-free environment of the animal facility of Taipei Medical University. The mice had free access to pelleted food (Labdiet^®^ 5010) and tap water at all times. All animals were maintained in compliance with the Guide for the Care and Use of Laboratory Animals Eight Edition published by the National Research Council of the National Academies. All experiments were approved by the Institutional Animal Care and Use Committee of Taipei Medical University (approved proposal no. LAC-2014-0217) and were performed in accordance with institutional guidelines. The mice were monitored daily for physiological signs. The growths of the tumor were monitored using micro-CT every week. Mice were anesthetized by inhalation of 5% isoflurane followed by 2% isoflurane for maintenance during an imaging process. Total lung volumes were analyzed and measured using software CTAn (v.1.15), mice were euthanized when total lung volumes were less than 120 mm^3^. After the investigation, tested animals were euthanized by 100% CO_2_ inhalation to minimize the animals suffering.

### Histology and Immunohistochemistry (IHC)

Tumors and mouse lungs were removed and stored in 10% neutral buffered formalin for paraffin embedding and sectioning. Paraffin-embedded tumor and lung samples were sectioned at 5 μm and stained with hematoxylin-eosin (Sigma-Aldrich). IHC staining was performed on 5-μm-thick, formalin-fixed, paraffin-embedded tissue sections. Paraffin-embedded tissue sections were deparaffinized (two changes of xylene for 5 min each, absolute ethanol for 3min, 75% ethanol for 3 min, and a deionized water rinse) before IHC staining. After antigen retrieval by microwave pretreatment in 10 mM citrate buffer, pH 6.0, for 10~20 min, sections were cooled and incubated with the primary antibody at 4°C overnight. Primary antibody dilutions were made in blocking buffer (10% bovine serum albumin with 0.1% Triton-100 in phosphate-buffered saline (PBS)) as follows: 1:100 anti-MRP3 (ab3376, Abcam) and 1:200 anti-TTF-1 (ab76013, Abcam). Immunochemical signals were detected using a MultiLink Detection Kit (Fremont). The peroxidase reaction was developed with diaminobenzidine, and sections were counterstained with Mayer's hematoxylin. Mucin staining was performed using a periodic acid-Schiff (PAS) staining kit following the manufacturer’s instructions (ab150680, Abcam).

### Micro-Computed Tomography (micro-CT)

Four male and female mice were scanned in the supine position using in vivo micro-CT (SkyScan 1176, Kontich, Belgium). Mice were sedated with isoflurane during the scan, and the breath rate was stable (0.7~0.9 breaths/s). The scan was performed using a resolution of 35 μm and was operated at a voltage of 50 kVp, a current of 500 μA, and an exposure time of 50 ms with a 0.5-mm aluminum filter. Images were taken using the synchronizing mode to prevent artifacts caused by cardiac and respiratory motion [20]. Sections were reconstructed using a GPU-based NRecon software. The region of interest was cropped and separated and was further analyzed by CTAn software (Bruker Skyscan). The air volume inside the lung area was separated and analyzed. The 3D data were calculated by CTAn software. Dataviewer software (Bruker Skyscan) was used to provide representative cross-sectional images.

### Microarray analysis

Total RNA (0.2 μg) was amplified by a Low Input Quick-Amp Labeling kit (Agilent Technologies) and labeled with Cy3 (CyDye, Agilent Technologies) during the in vitro transcription process. Cy3-labeled complementary (c)RNA (0.6 μg) was fragmented to an average size of about 50~100 nucleotides by incubation with fragmentation buffer at 60°C for 30 min. Corresponding fragmented, labeled cRNA was then pooled and hybridized to an Agilent SurePrint G3 Mouse GE 8 x 60K Microarray (Agilent Technologies) at 65°C for 17 h. After washing and drying by blowing with a nitrogen gun, microarrays were scanned with an Agilent microarray scanner (Agilent Technologies) at 535 nm for Cy3. Scanned images were analyzed, and normalization software was used to quantify the signal and background intensities of each feature. Microarray dataset of Tg-3m and Tg-6m tumors was deposited in public database Dryad (http://datadryad.org/; doi:10.5061/dryad.n01tb). Knowledge-based Gene Set Enrichment Analysis (GSEA) software was used to analyze the experimental datasets.

### Establishment of lung tumor cell lines

To establish primary lung tumor cell lines, lung tumors obtained from transgenic mice at various ages were processed within 30 minutes after sacrifice. Tumor specimens were rinsed twice with PBS supplemented with penicillin and streptomycin solution (P/S) (15140163, Gibco) and finely minced with scissors. Both necrotic tissue and apparently normal tissue were discarded. Tumor fragments were then immersed into DMEM medium supplemented with collagenase (C5138-100MG, Sigma) 1000 unit/ml, DNase (D4527-10KU, Sigma) 0.1mg/ml, hyaluronidase (H3757-100MG, Sigma) 300 unit/ml and P/S in 37°C incubator containing 5% CO_2_, followed by pipetting every 30 minutes for 2 hr. The cell suspension was then filtered through 100 μm sieve to remove tissue clumps followed by centrifuging at 300 g for 5 minutes. Cells were re-suspended in RPMI 1640 (containing 1.0 mg/ml of recombinant human epidermal growth factor (EGF) and 0.5 mg/ml of recombinant human basic fibroblast growth factor FGFb, Gibco) with 10% FBS and P/S and cultured in 37°C incubator containing 5% CO_2_. The medium was changed every 3–4 days. Once the cells were confluent, they were digested with 0.05% EDTA-trypsin for passage.

### Protein preparation and Western blotting

Proteins were extracted from cells using RIPA buffer containing protease inhibitors (P8340, Sigma), followed by sonicating cells for 30 s, then placing them on ice five times for 30 s each time. After sonication, cell extracts were centrifuged at 14,000 rpm for 10 min at 4°C. Supernatants were then collected and stored at -80°C. Protein concentrations were determined by a Bio-Rad Protein Assay Kit (Cat-500-0006). Total protein concentrations of 50 μg were boiled in dye buffer (10% sodium dodecyl sulfate (SDS), 0.25 M Tris-base, and 30% glycerol; pH 8.8) for 5 min at 95°C followed by electrophoresis using 8% SDS-polyacrylamide gel electrophoresis (PAGE). Gels were then transferred to a polyvinylidene difluoride membrane (NENTM, Life Science) using 1× Transfer Buffer (20 mM Tris, 150 mM glycine, 0.1% SDS, and 10% methanol) for 90 min at 120 V. Blots were then incubated for 1 h at room temperature with blocking buffer of 5% skimmed milk in TBST (150 mM NaCl, 10 mM Tris at pH 8.0, and 0/1% Tween 20) buffer. After blocking, blots were immunostained with antibodies as indicated (1:200 of anti-E-cadherin antibody, 610181, BD; 1:800 of anti-fibronectin antibody, ab23750, Abcam; 1:5000 of anti-GAPDH antibody, GTX100188, GeneTex). After incubation with HRP conjugated secondary antibody (1:2000 of goat anti-mouse IgG, ab6717, Abcam or 1:4000 of goat anti-rabbit IgG, GTX213110-01, GeneTex), protein bands were detected by an enhanced chemiluminescent (ECL) reagent.

### RNA isolation and RT-PCR analysis

Total RNA was extracted from tissue samples or cells using the TRIzol^®^ Reagent (Sigma) following instructions provided by the manufacturer. Three micrograms of total RNA were reverse-transcribed into cDNA using a Maxima First Strand cDNA Synthesis Kit for an RT-qPCR (K1671, Thermo). The RT-PCR was performed on an Eco™ Real-Time PCR System (Illumia) in a final reaction volume of 10 μl containing specific primers as indicated (S1 Table). GAPDH was used as an internal control. After incubation at 95°C for 10 min, the PCR was performed with 40 cycles of 95°C for 10 s, 58°C for 30 s, and 72°C for 1 min and the products were visualized by agarose gel electrophoresis.

### Three-Dimensional (3D) live-cell imaging

Holographic time-lapse imaging cytometry HoloMonitor M4 (Phase Holographic Imaging, Lund, Sweden) was used to capture 24-h time-lapse movies of Tg-3m and Tg-6m cells. Cells were seeded in a 6-well plate at a density of 10^5^ cells per well, allowed to adhere in RPMI 1640 (including 1.0 mg/ml of recombinant human epidermal growth factor (EGF) and 0.5 mg/ml of recombinant human basic fibroblast growth factor FGFb, Gibco) medium overnight, and then directly transferred to phase holographic microscopy. For each individual cell, the center of mass was determined and followed over time. Images were saved every 30 min for 24 h.

### Ingenuity Pathways Analysis (IPA)

The functions and roles in signaling pathways of genes obtained from the RNA expression arrays of Tg-3m and Tg-6m lung tumors and wild-type lung samples were analyzed using IPA (vers. 8.7, Ingenuity System, USA). IPA is an all-in-one, web-based software application that enables analysis, integration, and understanding of data from gene expression, micro (mi)RNA, and single-nucleotide polymorphism (SNP) microarrays, as well as metabolomics, proteomics, and RNAseq experiments. In this study, we utilized the IPA core analysis to identify biological functions and canonical pathways associated with cell movement, migration, and infiltration and generate relevant networks. The resulting candidate genes were further examined by an RT-qPCR from tissue and cell samples.

### Statistics

The statistical tests used in each analysis are stated in the corresponding Fig legends. Statistical analyses were performed using a *t*-test or one-way ANOVA depending on experimental groups. *P*-values are depicted using asterisks, with *P<0.05, **P<0.01 and ***P<0.001. Error bars in all Figs represent the standard deviation (s.d.).

## Results

### Histological analysis of spontaneous lung tumors reveals the presence of progressive lesions

**Fig 1:**
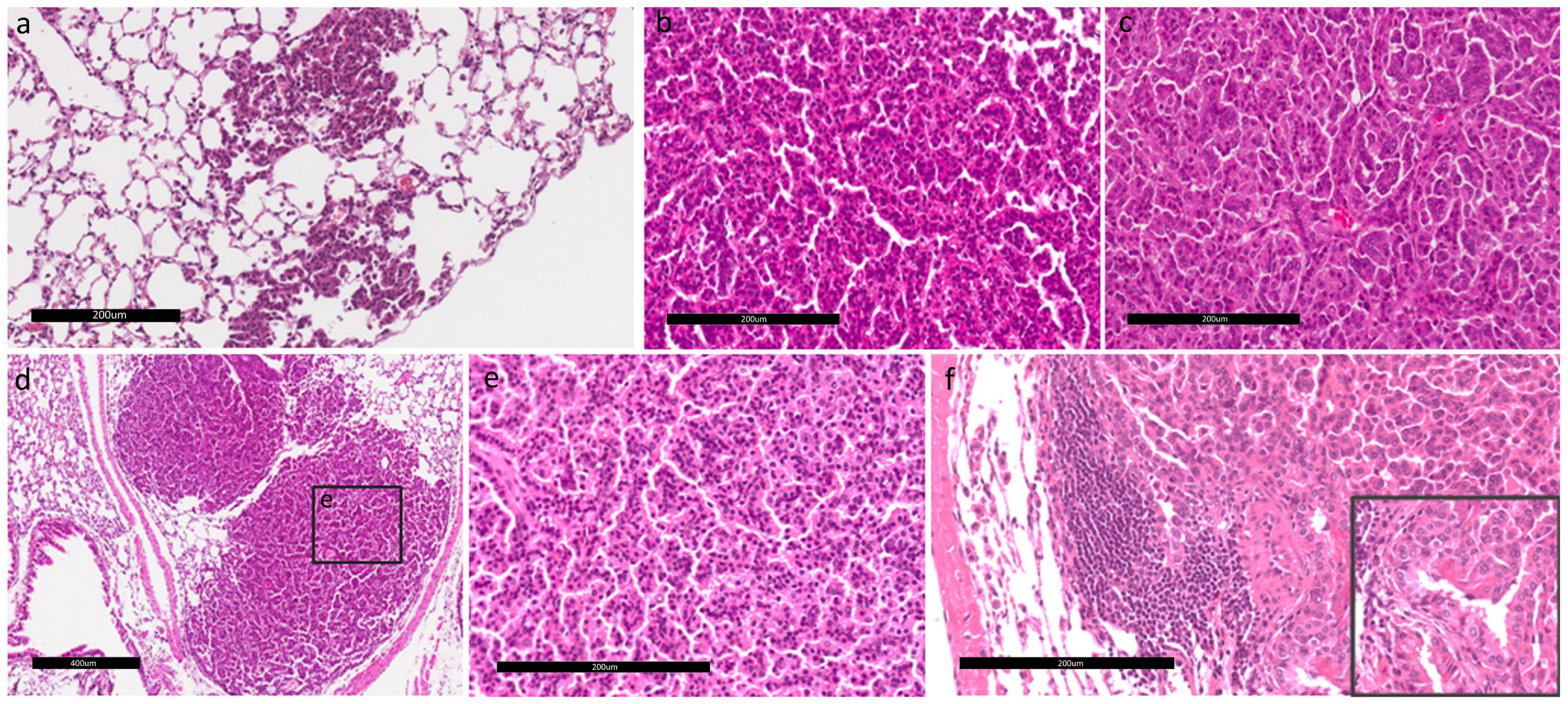
Tumor progression and histopathological phenotype in a transgenic mouse model. Hematoxylin and eosin (H&E)-stained tumors from Tg mice at various stages of age: (a) an atypical adenomatous hyperplasia (AAH) lesion progressing to adenocarcinoma *in situ* (AIS). The scale bar showed 200 μm in distance. (b) Uniform nuclei are shown in an AIS. The scale bar showed 200 μm in distance. (c) Pleomorphic nuclei are shown in an adenocarcinoma. The scale bar showed 200 μm in distance. (d) Formation of lung adenocarcinomas. The scale bar showed 400 μm in distance. (e) Adenocarcinoma with glandular/acinar architecture and desmoplasia. The scale bar showed 200 μm in distance. (f) Invasive adenocarcinoma displaying mixed cellular phenotypes. Cells of the invasive component were more columnar and of higher nuclear grade (inset). The scale bar showed 200 μm in distance. Panels a and d were at 100x magnification; panels b, c, e, and the inset were at 400x magnification; panel f was at 200x magnification.

Histological analysis of spontaneous lung tumors taken from the transgenic mouse at ages of 3 and 6 months onwards revealed the presence of tumor progression including AAH, AIS (which was previously called BAC), and ADC as illustrated in Fig 1. The term “tumor progression” here is used to describe the process of tumor development from lung adenomas to lung ADCs, which is closely associated with an increase in the total tumor load per mouse. AAH is defined as a localized proliferation of atypical epithelial cells growing along alveolar septa without disrupting the underlying lung architecture (Fig 1a). The AAH present in the transgenic mouse was observed at the early age and developed into AIS, eventually developing into an ADC over time. AAHs featured uniform nuclei, while the nuclear pattern became pleomorphic in AIS (Fig 1b and 1c). Transgenic mice at age of 6 months onwards developed ADCs featuring glandular/acinar architecture and desmoplasia showing an invasive phenotype (Fig 1d–1f).

### IHC analysis reveals lung tumors are related to ADCs

**Fig 2:**
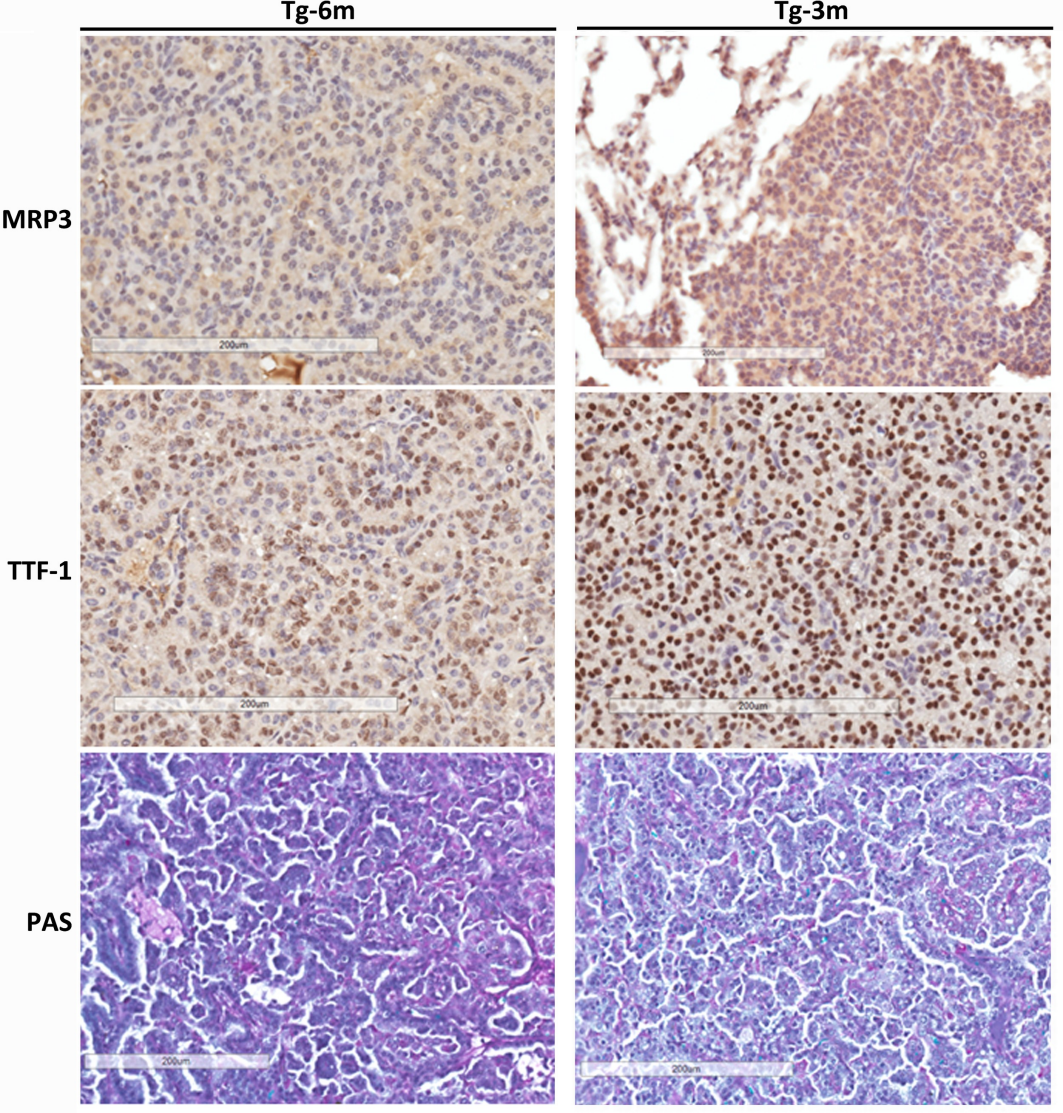
IHC analysis reveals lung tumors are displaying markers related to adenocarcinoma. IHC evaluation of multidrug resistant protein 3 (MRP3), thyroid transcription factor (TTF)-1, and periodic acid-Schiff (PAS) stain in tumors obtained at 6 (Tg-6m) and 3 months (Tg-3m). MRP3 expression was only diffused in Tg-3m lung tumors. Nuclei from tumor cells of both Tg-6m and Tg-3m were stained with TTF-1; it was more intense in Tg-3m than Tg-6m tumor cells. Both Tg-6m and Tg-3m tumor samples were PAS-positive. Panels are at 200x magnification. The scale bar showed 200 μm in distance.

IHC staining with MRP3 [21], TTF-1 [22], and PAS was applied to differentiate lung tumors obtained from transgenic mice at 3 and 6 months of age (Fig 2). MRP3, reported to be an early marker of lung ADC, was detected positively in 3-month (Tg-3m) tumor cells, but not in Tg-6m cells. TTF-1, a classic marker for ADCs, was expressed in both tumor stages. Both Tg-3m and Tg-6m tumor cells were also PAS-positive, which confirmed tumor differentiation as lung ADCs. Taken together, the immuno-profile of lung tumors developed in transgenic mice proved its relationship to human lung ADCs.

### Pulmonary tumor growth and metastasis of transgenic mice

**Fig 3:**
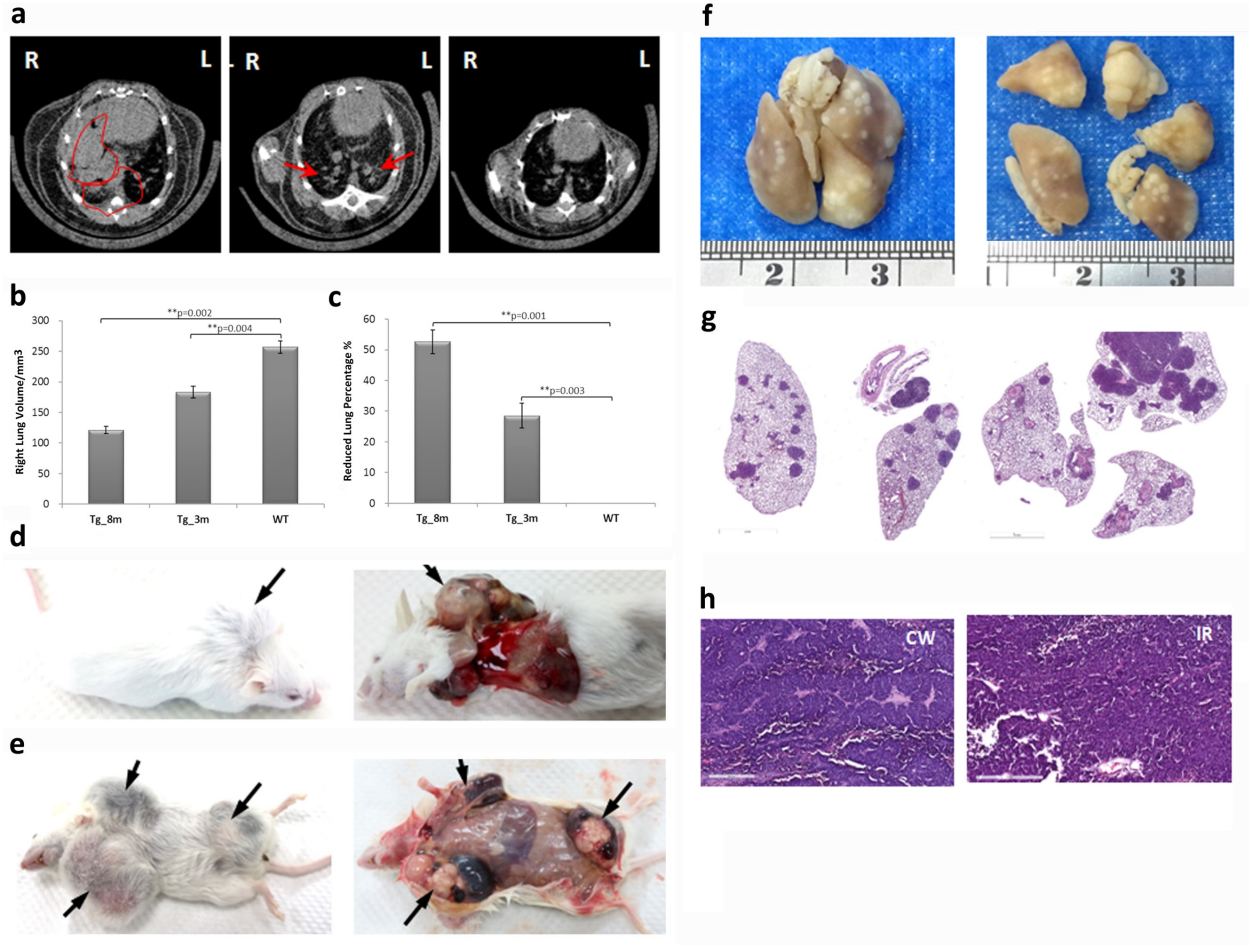
Tumor growths and metastasis of transgenic mice. (a) Normal lung (wild-type; WT) and lung tumors at 3 (Tg-3m) and 8 months (Tg-8m) were evaluated using micro-CT. Arrows and circles indicate where solitary tumor nodules were observed. (b-c) Bar charts represent analytical results of the growing lung volume (mm^3^) and the reduced lung volume percentage compared to wild-type mouse (%). Statistical analysis between each stage of lung tumors and wild-type lung (Tg-8m v.s. WT and Tg-3m v.s. WT) was significant (*P* value <0.05) in *t*-test; group statistics of Tg-8m, Tg-3m, and WT lungs were also significant (*P* value <0.001) as verified using ANOVA (data not shown). The lung images shown of each age were representative of more than 3 animals that been scanned. (d-e) Macroscopic features of tumors in the chest wall and inguinal region. (f) Macroscopic and histological (g) image of formalin-fixed lung tumor multiplicity (100x magnification, H&E stained). (h) Histological examination of metastatic tumors in the chest wall (CW) and inguinal region (IR) (H&E stained). Histology of the metastatic nodules revealed typical ADCs features of rounded aggregates of tumor cells or acinar pattern with a glandular formation and cribriform arrangements. Scattered tumor cells within myofibroblastic stroma were also noted (400x magnification).

Live micro-CT scanning was performed on transgenic mice starting from the age of 1 month at 2-week intervals to monitor tumor development. Primary lung cancers in mice, unlike many other tumor types, are challenging to image with high resolution due to cardiac and respiratory motion artifacts and small tumor sizes. Images in this study were taken using the synchronizing mode to prevent artifacts. Tumor growth was noted as an increase in tumor size and incidence by age compared to normal lungs (Fig 3a). The lung volume was measured by subtracting obstructive tumor nodules using CTAn software (Bruker Skyscan) (Fig 3b). As time progressed, the lung was occupied by growing tumors, and the lung volume was reduced by up to 50% by the age of 8 months (Fig 3c). Tumor metastasis was also noted in transgenic mice at the late stage. Fig 3d and 3e show the images of the gross anatomy of tumor-bearing transgenic mice where the metastatic nodules were visible at chest wall and inguinal region. Macro pathology and histopathology of formalin-fixed lung-derived from Fig 3e showed tumor multiplicity (Fig 3f and 3g). The histological images of the tumors in the chest wall (Fig 3h CW) and inguinal region (Fig 3h IR) was H & E stained and examined by a medical pathologist as typical ADCs features of rounded aggregates of tumor cells or acinar pattern with a glandular formation and cribriform arrangements. Scattered tumor cells within myofibroblastic stroma were also noted.

### Transgenic mice displayed differential genetic signatures and the EMT phenomenon

**Fig 4:**
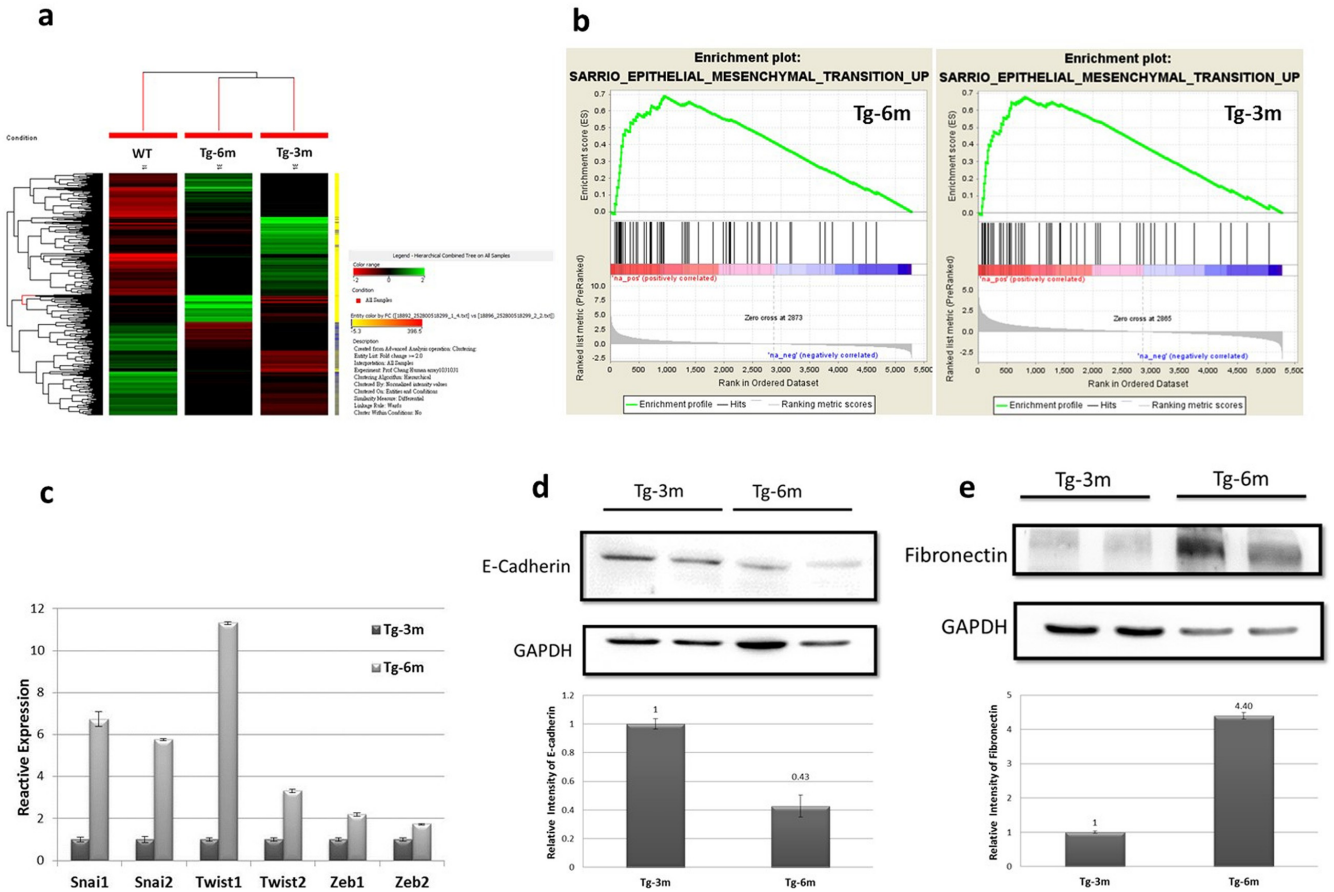
Six months (Tg-6m) mice displayed the epithelial-mesenchymal transition (EMT) phenomenon. (a) Hierarchical cluster analysis of normal lung and lung tumors from Tg-3m and Tg-6m mice. (b) GSEA data showing enrichment of EMT signatures in both Tg-6m and Tg-3m tumors. (c) Real-time qPCR of key EMT regulators, *Snail*, *Twist*, and *Zeb*, indicates they were upregulated in Tg-6m tumor cells. Data shown were representative of 3 independent experiment. (d, e) Western blot analysis of E-cadherin and fibronectin proteins in Tg-3m and Tg-6m tumor cells. The amount of the epithelial marker, E-cadherin, had decreased in Tg-6m cells compared to Tg-3m cells (d). In contrast, the amount of the mesenchymal marker, fibronectin, had increased in Tg-6m cells (e). Shown were representative blots of 3 independent experiments.

Analyses of lung tumors from different ages of transgenic mice compared to age-matched normal lung sample were performed using a cDNA array technique. A hierarchical cluster analysis of 2624 genes (with at least 2 multiples of change) was illustrated and grouped according to functional pathways (Fig 4a). The unsupervised agglomerative hierarchal cluster analysis of differentially expressed genes within lung tumors and normal lungs demonstrated significant differences in gene expressions. To evaluate whether tumors from transgenic mice displayed global EMT-related changes, we performed gene expression profiling of tumor cells derived from Tg-3m and Tg-6m mice. A GSEA indicated that a distinct EMT signature was significantly enriched in both cell lines, strongly suggesting that the EMT phenomenon occurred in transgenic mice (Fig 4b). Furthermore, with analysis by a real-time qPCR, key transcriptional factors of the EMT such as *Snail1*, *Snail2*, *Twist1*, *Twist2*, *Zeb1*, and *Zeb2*, were found to be markedly expressed in Tg-6m tumor cells when normalized to Tg-3m tumor cells (Fig 4c and S3 Table). In agreement with the upregulation of EMT-associated transcription factors, protein levels of the epithelium marker, E-cadherin, were 6-fold reduced in Tg-6m compared to Tg-3m tumor cells (Fig 4d). In contrast, expression of the mesenchymal marker, fibronectin, was at least 4-times upregulated in Tg-6m compared to Tg-3m tumor cells (Fig 4e). Collectively, these data suggested that tumors in our mouse model displayed the EMT phenomenon after 6 months of age.

### Tg-6m cells have higher flexibility and mobility

**Fig 5:**
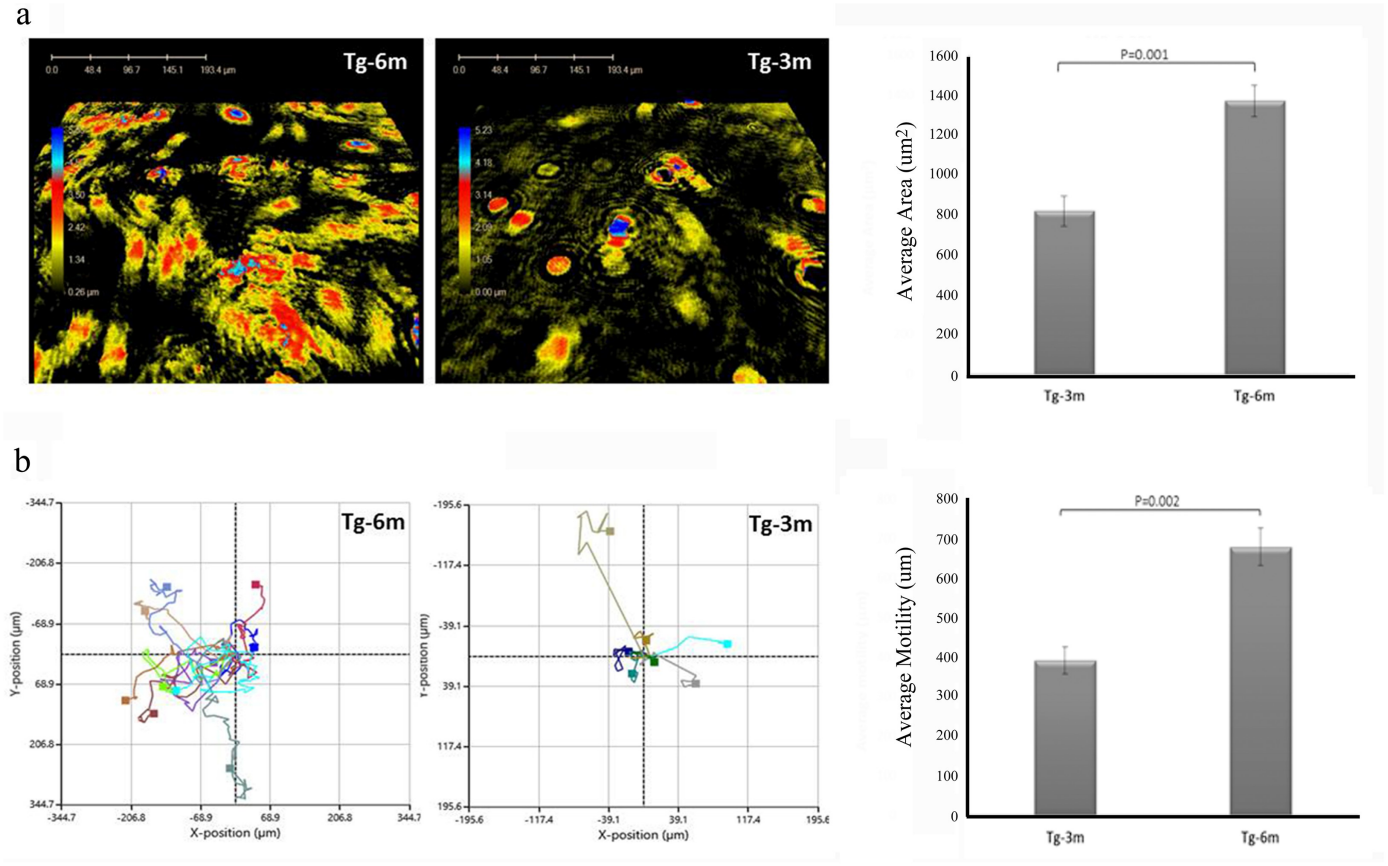
Holographic microscopy analysis of cellular movement. Six-month (Tg-6m) cells were more versatile compared to Tg-3m cells in the captured image. Surface areas of Tg-3m and Tg-6m cells were measured and statistically plotted in a bar chart (μm^2^). (b) The paths of cell migration are presented as individual colored lines from Tg-3m and Tg-6m cells, and the average motility was calculated in the bar chart (μm). The Fig showed representative results of 3 independent experiments.

We next investigated the capability of cell movement of tumor cells at different stages. Using phase holographic microscopy, we captured 24-h time-lapse movies of cells (S1 and S2 movies). The center of mass of each individual cell was determined and followed over time. As shown in the results, cells became more elongated and versatile in shape in Tg-6m with an increased surface area compared to Tg-3m cells (Fig 5a). The resulting traces from both types of cells showed that Tg-6m cells moved more vigorously than Tg-3m cells (Fig 5b).

### Identification of novel EMT-relevant genes involved in lung ADC development

**Fig 6:**
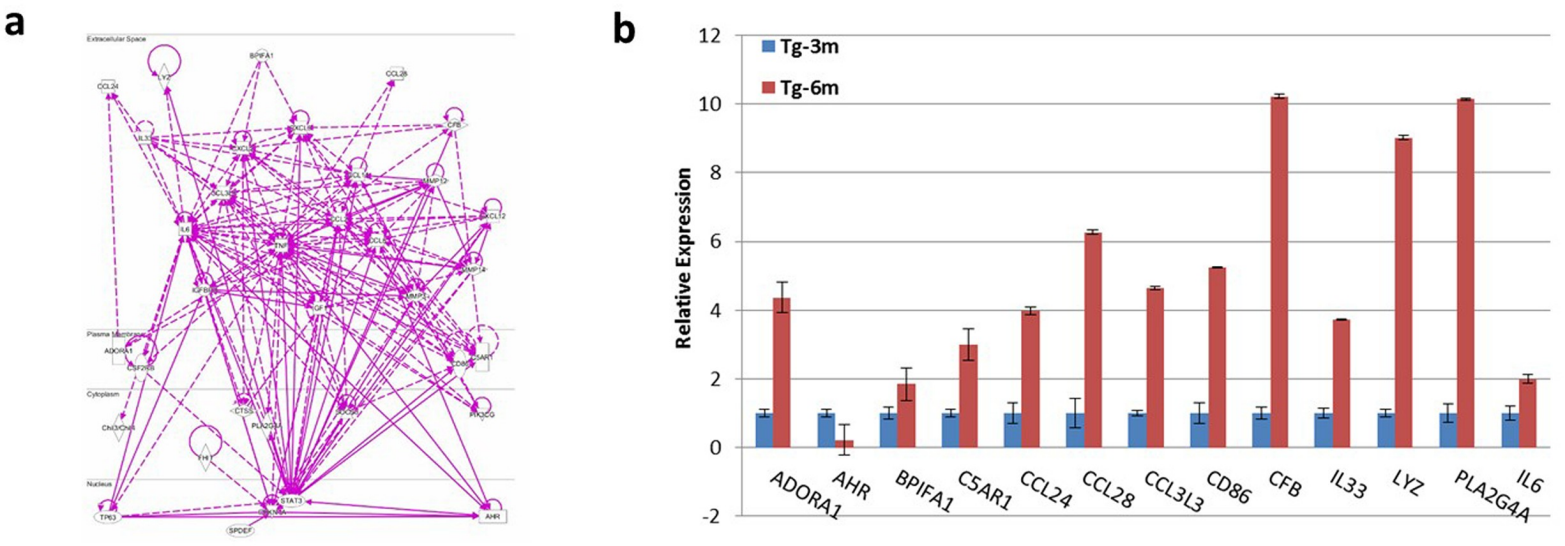
Candidate genes associated with the epithelial-mesenchymal transition (EMT). (a) IPA-analyzed EMT-relevant genes from 6 months (Tg-6m) lung tumors and their interactive network according to cellular locations. (b) Expression levels of EMT-related genes from Tg-3m and Tg-6m tumor cells were examined using a real-time qPCR. Data shown were representative of 3 independent experiment.

Changes in gene expressions that contributed to repression of the epithelial phenotype and activation of the mesenchymal phenotype involved master regulators, including SNAIL, TWIST, and ZEB transcription factors, as shown in Fig 4. We further analyzed bioinformatics information obtained from the expression array using IPA in the category of cellular movement and migration, which resulted in a number of candidate genes that were less documented in the association with EMT (S2 Table) and an interactive network according to the cellular location (Fig 6a). Despite EMT-related genes being well studied, a real-time qPCR was performed to screen expression levels of candidate genes from Tg-3m and Tg-6m tumors compared to wild-type mouse lung and several genes of interest were identified (Fig 6b and S4 Table). We also used MaraCards, a comprehensive human disease compendium, to evaluate the occurrences of these genes in human lung cancer. A MalaCards Information Score (MIFTS) is assigned to define the richness of information for each disease, and the score ranges 1~101. Collectively, from the bioinformatics information, we obtained four genes, *LYZ*, *BPIFA1*, *CFB*, and *AHR*, which were found worthy of further investigation both from IPA and MalaCards. Among these, *LYZ*, *BPIFA1*, and *CFB* were noted to be upregulated in Tg-6m cells, while *AHR* was found to be downregulated. Further experiments are required to investigate the roles of *LYZ*, *BPIFA1*, and *CFB* in the progression and development of lung ADCs and to confirm the repressive role of *AHR*.

## Discussion

In this study, we characterized an established transgenic mouse model with spontaneous lung tumors in terms of the histopathology, transcriptome profiles, and their respective molecular signatures in human lung ADCs. Major limitations of existing murine lung cancer models include the inability to detect the early onset and the lack of overall genetic profiles regarding tumorigenesis. We provide evidence to show that our transgenic mice spontaneously developed lung tumors and displayed an early marker (MRP3) of lung ADC and the classic markers of TTF-1 and PAS. In addition, the tumor size and incidence could be monitored using micro-CT over a long time span (more than 1 year). This may more closely mimic human lung cancer, in which patients typically develop a single lung tumor that progresses over time to metastatic disease.

IPA was adapted to analyze results from the expression array derived from Tm-3m and Tm-6m lung tumors. Genes related to the category of the cellular movement were obtained and sorted by a literature review to retain those that were novel to lung metastasis for a further RT-qPCR analysis. As a result, we identified 13 functionally related genes that are associated with cell movement/migration and are expressed in tumors. Among the 13 candidate genes related to metastasis, *LYZ*, *BPIFA1*, *CFB*, and *AHR* were identified to have high scores in MalaCards for human lung cancer. These genes were found to play roles in immune regulation, either in activation of innate responses or complement.

LYZ is a classic macrophage marker. Tumor-associated macrophages (TAMs) are some of the major constituents of tumor stroma in many solid tumors, and there is compelling preclinical and clinical evidence that macrophages promote cancer initiation and malignant progression [23]. Recently, it was also reported that TAMs interact with cancer stem cells, which facilitate tumorigenicity, metastasis, and drug resistance [24]. The *BPIFA1* (also known as *SPLUNC1*, *LUNX*, or *PLUNC*) protein is predominantly expressed in the epithelium of the upper respiratory tract, including the nasopharynx, and functions in the innate immune response against infections by a variety of pathogens [25]. BPIFA1 is thought to be involved in inflammatory responses to irritants in the upper airways and may also serve as a potential molecular marker for detecting micrometastases in NSCLC. *CFB* encodes complement factor B, a component of the alternative pathway of complement activation. Recent research of complements demonstrated several interconnections between the complement system and cancer. Despite being in accordance with our traditional understanding of complement functions in immunity, the discovery that complement C5a promotes tumor growth challenges the dogma that enhancement of complement activation is beneficial for cancer patients [26].

Among the candidate genes identified, the expression of *AHR* was found to have been markedly decreased in Tg-6m tumors. The aryl hydrocarbon receptor (AhR) is a ligand-activated transcription factor that has been extensively studied as a mediator of toxicant metabolism; however, increasing evidence has demonstrated that the AhR plays an important role in immune processes [27]. AhR signaling plays a key role in immunological functions at mucosal surfaces, including the lungs. Experiments using AhR-deficient mice showed that they developed heightened inflammatory responses in the lungs [27]. The reduced expression of *AHR* in our results suggests an association of increased inflammation and the progression of lung ADCs.

It is well established that the tumor microenvironment is rich in inflammatory mediators such as cytokines and chemokines, which facilitate the proliferation of transformed cells, thereby contributing to the process of cancer promotion [28]. Collectively, we provide supportive evidence to characterize the transgenic mouse model with spontaneous lung ADCs to its human counterpart, which makes drug evaluation and biomarker screening possible. Identification of *LYZ*, *BPIFA1*, *CFB*, and *AHR* genes in our transgenic mouse model suggests high involvement of inflammatory mediators in the progression of lung ADCs. Chronic inflammation seems to play a major role in the onset and development of cancer. In this study, we highlight the involvement of the innate immunity, such as macrophages and complements, in the carcinogenesis of lung ADCs. Understanding interactions between cellular and molecular mechanisms that mediate inflammation in lung ADCs will help elucidate novel targets affecting key oncogenic pathways in this malignancy and allow prevention of cancer cell migration and metastasis.

## Supporting information

S1 Movie 24-h time-lapse movies of Tg-3m cells. (AVI)

S2 Movie 24-h time-lapse movies of Tg-6m cells. (AVI)

S1 Table qRT-PCR primer sequences of EMT transcriptional factors and candidate genes. (PDF)

S2 Table IPA analyzed genes related to cellular movement and migration. (PDF)

S3 Table Statistics of key transcriptional factors of EMT from real-time qPCR of Tg-3m and Tg-6m cell lines. (PDF)

S4 Table Statistics of candidate genes associated with EMT from real-time qPCR of Tg-3m and Tg-6m cell lines. (PDF)

## Abbreviations

AAH: atypical adenomatous hyperplasia
ADC: adenocarcinoma
AIS: adenocarcinoma *in situ*
BAC: bronchoalveolar carcinoma
EMT: epithelial-mesenchymal transition
Env: envelope protein
HERV: human endogenous retrovirus
HPV: human papilloma virus
IPA: ingenuity pathway analysis
JSRV: jaagsiekte sheep retrovirus
MRP3: multidrug resistant protein 3
NSCLC: non-small-cell lung cancer
OPA: ovine pulmonary adenocarcinoma
PAS: periodic acid-Schiff stain
SCC: squamous cell carcinoma
SCLC: small-cell lung cancer
SPC: surfactant protein C
TTF-1: thyroid transcription factor-1
WHO: World Health Organization
