# Supplementary material for: Characterization of a transgenic mouse model exhibiting spontaneous lung adenocarcinomas with a metastatic phenotype
Source: PLoS One. 2017 Apr 18;12(4):e0175586. doi: 10.1371/journal.pone.0175586 (PMC5395147; doi:10.1371/journal.pone.0175586)
Supplement: S1 Table — (PDF) [file pone.0175586.s003.pdf]

S1 Table

qRT-PCR primer sequences of EMT transcriptional factors and candidate genes

Gene	Primer
SNAIL1	F: ctggtgagaagccattctcc<br>R: ggaagatgccagcgaggatg
SNAIL2	F: cctggctgcttcaaggacac<br>R: cactcagtgtgccacacagc
TWIST1	F: ctggactccaagatggcaag<br>R: ccagagtctctagactgtcc
TWIST2	F: gatgaccagctgcagctacg<br>R: ccacaaggttgtccaggtgc
ZEB1	F: ccagtgaaggtgatccagcc<br>R: gaggcctcttacctgtgtgc
ZEB2	F: gctcctactcgcagcacatg<br>R: cctccttctcgtgctccttc
ADORA1	F: ctattcacagcctgggactc<br>R: ccaagggttgtacttcctgg
AHR	F: ctccctgtaatcagcctctg<br>R: ctgatacatggacatggccc
BPIFA1	F: ctctgggcttgacactcaac<br>R: caggagtgactccattgagc
C5AR1	F: catctgctacaccttcctcc<br>R: cacagggagttcagcttctc
CCL11	F: gggaatatcagcaccagtcg<br>R: ctcgtcccattgtgttcctc
CCL24	F: ctctgtccctgaacttggac<br>R: cacaggagacaccagaactc
CCL28	F: gagctagctctacatcccag<br>R: gccaggtttctcagtgctag
CD86	F: cagagtgaaggcagtgagag<br>R: gatgtctgctcctggcatag
CFB	F: gtctgatgagaggagtagcg<br>R: cgggttctattccagccttc
IL33	F: gacttcagaagagcaggcac<br>R: gtcagtctttgcagcagctg

LYZ1	F: ctactgcagcccattctgtc<br>R: ctgtgttggctgactgtcac
PLA2G4A	F: cagctccgacagtgatgatg<br>R: gctcgtcatcgaaggaatcc
GAPDH	F: cctggccaaggtcatccatg<br>R: cagctctgggatgaccttgc
