# Supplementary material for: Characterization of a transgenic mouse model exhibiting spontaneous lung adenocarcinomas with a metastatic phenotype
Source: PLoS One. 2017 Apr 18;12(4):e0175586. doi: 10.1371/journal.pone.0175586 (PMC5395147; doi:10.1371/journal.pone.0175586)
Supplement: S2 Table — (PDF) [file pone.0175586.s004.pdf]

S2 Table

IPA analyzed genes related to cellular movement and migration.

Symbol	Entrez Gene Name	Exp Log Ratio	MIFTS* of Lung Cancer
ADORA1	adenosine A1 receptor	-1.367	ND
PLA2G4A	phospholipase A2, group IVA (cytosolic, calcium-dependent)	-1.262	ND
AHR	aryl hydrocarbon receptor	-1.036	94
LYZ	lysozyme	1.011	94
STAT3	signal transducer and activator of transcription 3	1.014	94
TP63	tumor protein p63	1.043	94
CCL24	chemokine (C-C motif) ligand 24	1.054	ND
C5AR1	complement component 5a receptor 1	1.074	ND
CXCL12	chemokine (C-X-C motif) ligand 12	1.083	94
FHIT	fragile histidine triad	1.166	94
CCL11	chemokine (C-C motif) ligand 11	1.185	ND
CTSS	cathepsin S	1.226	ND
MMP14	matrix metallopeptidase 14 (membrane-inserted)	1.237	ND
CCL5	chemokine (C-C motif) ligand 5	1.243	94
PIK3CG	phosphatidylinositol-4,5-bisphosphate 3-kinase, catalytic subunit gamma	1.244	ND
SOCS3	suppressor of cytokine signaling 3	1.278	94
CD86	CD86 molecule	1.301	ND
IGFBP3	insulin-like growth factor binding protein 3	1.324	ND
IL33	interleukin 33	1.436	ND
CSF2RB	colony stimulating factor 2 receptor, beta, low-affinity (granulocyte-macrophage)	1.469	47 (pulmonary alveolar proteinosis)
IGF1	insulin-like growth factor 1 (somatomedin C)	1.784	94
SPDEF	SAM pointed domain containing ETS transcription factor	1.791	32 (lung cancer suceptibility 3)

CDKN1A	cyclin-dependent kinase inhibitor 1A (p21, Cip1)	1.800	94
CCL2	chemokine (C-C motif) ligand 2	1.861	94
BPIFA1	BPI fold containing family A, member 1	2.224	94
IL6	interleukin 6	2.416	ND
CCL28	chemokine (C-C motif) ligand 28	2.892	ND
TNF	tumor necrosis factor	3.027	94
MMP3	matrix metallopeptidase 3	3.171	ND
CXCL2	chemokine (C-X-C motif) ligand 2	3.274	ND
CFB	complement factor B	3.404	94
CCL3L3	chemokine (C-C motif) ligand 3-like 3	4.209	ND
MMP12	matrix metallopeptidase 12	6.577	94
CXCL3	chemokine (C-X-C motif) ligand 3	10.685	ND
