# Supplementary material for: Characterization of a transgenic mouse model exhibiting spontaneous lung adenocarcinomas with a metastatic phenotype
Source: PLoS One. 2017 Apr 18;12(4):e0175586. doi: 10.1371/journal.pone.0175586 (PMC5395147; doi:10.1371/journal.pone.0175586)
Supplement: S3 Table — (PDF) [file pone.0175586.s005.pdf]

S3 Table

Statistics of key transcriptional factors of EMT from real-time qPCR of Tg-3m and Tg-6m cell lines.

Genes	Average Cq (triplicate) Tg-3m	Tg-6m	SD	P value
Snail1	30.16	27.64	1.78	**p<0.01
Snail2	23.09	17.18	4.18	***p<0.001
Twist1	26.47	20.34	4.33	***p<0.001
Twist2	25.32	20.96	3.08	***p<0.001
Zeb1	21.4	17.64	2.66	***p<0.001
Zeb2	24.9	21.48	2.42	***p<0.001
