# Supplementary material for: Characterization of a transgenic mouse model exhibiting spontaneous lung adenocarcinomas with a metastatic phenotype
Source: PLoS One. 2017 Apr 18;12(4):e0175586. doi: 10.1371/journal.pone.0175586 (PMC5395147; doi:10.1371/journal.pone.0175586)
Supplement: S4 Table — (PDF) [file pone.0175586.s006.pdf]

S4 Table

Statistics of candidate genes associated with EMT from real-time qPCR of Tg-3m and Tg-6m cell lines.

	Average Cq (triplicate)			
**Genes**	**Tg-3m**	**Tg-6m**	**SD**	**P value**
ADORA1	29.55	24.45	3.61	***p<0.001
AHR	24.06	23.24	0.58	***p<0.001
BPIFA1	34.16	30.3	2.73	**p<0.01
C5AR1	28.28	23.73	3.22	***p<0.001
CCL24	32.07	28.28	2.68	***p<0.001
CCL28	28.61	24.16	3.14	**p<0.01
CCL3L3	35.83	30.1	4.05	*p<0.05
CD86	30.85	26.66	2.96	**p<0.01
CFB	28.61	23.47	3.64	***p<0.001
IL33	21.02	17.4	2.56	***p<0.001
LYZ	28.45	23.55	3.46	***p<0.001
PLA2G4A	23.38	18.31	3.59	***p<0.001
IL6	27.65	24.16	2.47	**p<0.01
